# A Two-Phase Distributed Filtering Algorithm for Networked Uncertain Systems with Fading Measurements under Deception Attacks

**DOI:** 10.3390/s20226445

**Published:** 2020-11-11

**Authors:** Raquel Caballero-Águila, Aurora Hermoso-Carazo, Josefa Linares-Pérez

**Affiliations:** 1 Departamento de Estadística, Universidad de Jaén, Paraje Las Lagunillas, 23071 Jaén, Spain; raguila@ujaen.es; 2 Departamento de Estadística, Universidad de Granada, Avda. Fuentenueva, 18071 Granada, Spain; ahermoso@ugr.es

**Keywords:** networked uncertain systems, fading measurements, distributed filtering, random deception attacks

## Abstract

In this paper, the distributed filtering problem is addressed for a class of discrete-time stochastic systems over a sensor network with a given topology, susceptible to suffering deception attacks, launched by potential adversaries, which can randomly succeed or not with a known success probability, which is not necessarily the same for the different sensors. The system model integrates some random imperfections and features that are frequently found in real networked environments, namely: (1) fading measurements; (2) multiplicative noises in both the state and measurement equations; and (3) sensor additive noises cross-correlated with each other and with the process noise. According to the network communication scheme, besides its own local measurements, each sensor receives the measured outputs from its adjacent nodes. Based on such measurements, a recursive algorithm is designed to obtain the least-squares linear filter of the state. Thereafter, each sensor receives the filtering estimators previously obtained by its adjacent nodes, and these estimators are all fused to obtain the desired distributed filter as the minimum mean squared error matrix-weighted linear combination of them. The theoretical results are illustrated by a simulation example, where the efficiency of the developed distributed estimation strategy is discussed in terms of the error variances.

## 1. Introduction

In recent years, the signal estimation problem in multi-sensor systems has gradually become a meaningful topic of research, on account of its great significance in different applied and theoretical fields, such as space and terrestrial exploration, access in hazardous environments, factory automation, remote diagnostics and troubleshooting, domestic robots, experimental facilities, automobiles, aircraft, and manufacturing plant monitoring. It is well known that networked systems may often undergo random imperfections and disturbances (for example, missing and fading measurements, uncertainties of multiplicative noises, random delays, packet dropouts, and so on), which, if not addressed properly, are likely to impair the performance of the estimators. For this reason, considerable research efforts have been focused on the analysis of mathematical models involving these network-induced random phenomena and the design of estimation algorithms that do not neglect their effects (see, e.g., [1,2,3,4] and the references therein).

One of the most frequent uncertainties in networked systems is the fading or degradation of the measurements, which can be caused by potential restrictions of the physical equipment, the aging of the sensors, or the inaccuracy of the measurement devices, among others. This alludes to the cases when the network communication is not always perfect, so the system measurement fades/degrades in a random way, and it covers the missing measurement phenomenon as a particular case. The state estimation problem from fading measurements has attracted much research interest in the last few years. A recursive filtering algorithm for nonlinear stochastic systems with correlated noises, random parameter matrices, and fading measurements was proposed in [5]. The nonlinear filtering problem over sensor networks with fading channels was analyzed in [6] using the unscented Kalman filter methodology. The sequential fusion filtering problem was investigated in [7] for multi-sensor systems with fading measurements. The centralized and distributed filtering problems were addressed in [8] and [9], respectively, for discrete-time linear systems with fading measurements and time-correlated measurement noises, by using the measurement differencing method. Recently, a more general scenario involving time-correlated fading channels was considered in [10], where a recursive linear filtering algorithm was designed under the assumption that the random variables modeling the fading phenomenon obey a discrete-time uncertain system with random disturbances.

Another common type of uncertainty in networked environments is the presence of multiplicative stochastic disturbances in the system model, owing to different reasons, such as random failures and repairs of the components, changes in the interconnections of subsystems, sudden environment changes, and modification of the operating point of the model. Many research efforts have been made to design estimation algorithms that compensate the effects of these multiplicative noises in the system state and measurement equations. Optimal linear estimators in the minimum variance sense for discrete-time linear systems with random delays, packet dropouts, and white multiplicative noises were investigated in [11]. Centralized and distributed fusion filtering algorithms for multi-sensor systems with correlated white multiplicative noises in the state and measurement equations were designed in [12]. Furthermore, centralized and distributed fusion filters were designed in [13] for multi-sensor systems from measured outputs with both multiplicative and additive noises, assuming correlated random delays in transmissions. The state estimation problem for a class of discrete-time stochastic systems with multiplicative noises and unknown inputs over fading channels was addressed in [14]. The optimal linear estimation problem for uncertain systems in the presence of multiplicative noises and time-correlated additive noises was studied in [15], for a class of networked systems with fading measurements, and more recently, in [16], where uncertain systems with correlated white multiplicative noises and packet dropouts were considered and a sufficient condition for the convergence of the proposed estimators was established.

Despite their unquestionable advantages, sensor networks are also prone to have some vulnerabilities, which must be taken into consideration when solving the estimation problem to guarantee the accuracy of the designed estimators. Cyber-attacks are becoming one of the most popular vulnerabilities that reduce the reliability of a network. In the existing literature, the most typical kinds of cyber-attacks affecting the estimation and control problems can be broadly classified into three categories: availability attacks, replay attacks, and deception attacks [17,18]. The availability attacks, also called denial-of-service (DoS) attacks, prevent the data packet information from being sent by blocking or interrupting the communication channels (see, e.g., [17]). In the replay attack model, the adversaries hijack the sensors, capture the information, and forward it afterwards, in order to degrade the system performance (see, e.g., [19]). The last kind of cyber-attack is the so-called deception attacks, which compromise data integrity by directly modifying the measurement signal in a deliberate way. The complexity and significance of investigating this kind of attack have inspired many fruitful research efforts. In [20], an integrated design approach to simultaneously address the fault detection and fault estimation problems in discrete-time stochastic systems with event-triggered transmission, subject to unknown disturbances and randomly occurring deception attacks, was proposed. The remote state estimation in a multi-sensor framework was considered in [21], where the problem of detection against deception attacks was addressed. The distributed H${}_{\infty }$-consensus filtering problem for discrete-time systems with multiplicative noises and deception attacks over sensor networks was studied in [22]. A cluster-based approach was used in [23] to address the distributed fusion estimation problem for multi-sensor networked systems, when the measurements are subject to stochastic deception attacks, and the influence of unbounded false data injection attacks on state estimation processes was studied in [18].

The network connectivity and the communication scheme among sensors are some of the essential features to be accounted for in the design of estimation algorithms. Networked systems with a fixed topology represented by a directed graph are characterized by the cooperative mechanism, since the information available to be processed on each individual node is not only from its own measurements, but also from its neighboring sensor measurements according to the network topology. In the past few years, a great number of results have analyzed the distributed filtering problem in this kind of system with information communication among nodes. A class of networked systems with a fixed topology and multiplicative noises was considered in [24], where the distributed recursive filtering problem was investigated under the effects of uniform quantizations and deception attacks. Considering a fixed topology represented by a directed graph, the distributed filtering problem in networked multi-sensor systems with uncertainties modeled by random parameter matrices and correlated noises was investigated in [25]. Furthermore, in the case that the network topology among senor nodes is described by a directed graph, the event-based distributed state estimation problem for a linear system subject to unknown inputs and false data injection attacks was addressed in [26]. Other recent advances in the distributed filtering problem, considering different frameworks and assumptions for the system model, were reported in [27,28,29,30,31], just to mention a few.

Even though the random disturbances of the state and measurement models are frequently assumed to be uncorrelated with each other, this is not a realistic assumption in many practical applications, where the measurement noises are state dependent and, as a result, the process noise and the sensor noises, as well as the different sensor noises are cross-correlated. Furthermore, noise correlation unavoidably appears, for example when the state process is observed by sensors that operate in a common noisy environment, as well as in the augmented systems typically used to deal with random delays and packet dropouts. Regarding the correlation between the additive noises of the state and measurement equations, the estimation problem was addressed in [7] assuming that the sensor noises are correlated with the system noise at the same time step. In [32], it was considered that the measurement noise is correlated with the system noise at the previous time step; this kind of correlation arises, for example, in the linear systems obtained from the discretization of continuous-time systems, causing, at any time step, the measurement noises to be correlated with the signal at the same and subsequent time steps [33]. However, despite its practical significance, just some occasional results are reported on the subject of the distributed filter design problem in sensor networks with a fixed topological structure, in the presence of correlated noises (see, e.g., [34] and the references therein).

Research motivation: The above discussion leads us to the conclusion that, even though there exists a vast literature on the estimation problem over sensor networks, there are still several interesting challenges related to this topic, and this fact stimulates the main motivation of our research. Actually, in this paper, a comprehensive system model incorporating some of the most common above-listed random phenomena appearing in real networked environments (multiplicative noises, fading measurements, and deception attacks) is considered under the assumption that the process noise is correlated with the sensor noises, which, in turn, are cross-correlated with each other. These important aspects are dealt with in a unified yet effective framework to address the distributed filtering estimation problem over a sensor network with a fixed topological structure represented by a digraph. Due to the fact that the sensor network may not be fully connected and the use of zeros to weight those measurements from non-connected sensors is commonly handled by using generalized inverses when designing the filter and analyzing its performance, one of the main inherent difficulties that arise and that we purpose to overcome in this paper is the desirable avoidance of pseudo-inverse matrices in the computation process.

Main contributions: The main contributions of the current paper are summarized as follows: (1) the minimum mean squared error distributed filter is constructed for multisensor systems simultaneously covering fading measurements, the presence of multiplicative noises, and correlation in additive noises, subject to deception attacks; (2) a matrix simplification method is employed to avoid the use of pseudo-inverse matrices; (3) unlike other existing papers on distributed estimation, where an upper bound for the estimation error covariance is designed, in this paper, an exact expression of the filtering error covariance is obtained, with the additional advantage that it can be calculated off-line, thus providing an exact measure of the estimation accuracy that is not affected by the particular set of measurements being processed.

Related work: Some of the most closely related papers in the literature are [9,14,23,25]. In [9], discrete-time linear systems with fading measurements over connected networks were considered and distributed filtering estimators with a specific structure were obtained, where the gain matrices were determined using the minimum variance criterion. In contrast with the model in [9], multiplicative noise uncertainties in both the state and measurements, together with the presence of stochastic deception attacks, are covered by the model in the current paper; moreover, optimal linear distributed filtering estimators are designed using the mean squared error criterion, without imposing any structure on the estimators. A discrete-time stochastic system model including multiplicative noise in the state transition equation and random fading of the measurements was considered in [14]. In addition, the system model considered in the current paper incorporates multiplicative noise perturbations in the measurement equation, as well as random deception attacks. Furthermore, the estimation methodology is substantially different in both papers: a centralized Kalman filter was designed in [14], while least-squares distributed filtering estimators over a sensor network with a given topology are proposed in the current paper. In [23], networked systems subject to stochastic deception attacks, where the sensor nodes are grouped into clusters, were considered. Assuming that the sensors within each cluster are fully connected, least-squares linear distributed fusion estimators were obtained based on the measurements received from all the sensors in the same cluster. The main difficulty to address the distributed estimation problem in the current paper, compared to [23], was due to the fact that the sensor network was not fully connected, and the use of zeros to weight those measurements from non-connected sensors was commonly handled by using generalized inverses. However, as previously indicated, in this paper, an alternative matrix simplification methodology is employed to avoid the use of pseudo-inverses. Finally, the main difference between the system model in [25] and the current one is the presence of deception attacks. It is also remarkable that the derivation of the distributed filtering algorithm in [25] was based on the state-space model equations, while the algorithms proposed in the current paper do not need the explicit information provided by the state transition equation, but only the factorization of the state covariance matrix in a separable form.

Outline: The remainder of the paper is structured as follows. In Section 2, the linear uncertain system model with fading measurements and correlated noise, subject to deception attacks, is described. In Section 3, the distributed filtering problem is stated, the design phases of the algorithm are detailed, and the problem is reformulated in terms of a new gathered measurement model. Section 4 presents the proposed distributed filtering approach. Simulation results are shown in Section 5, and the conclusions are outlined in Section 6.

Notation: As much as possible, we will adhere to the conventional mathematical notation. The *n*-dimensional Euclidean space will be denoted as $R^{n}$, while $R^{m\times n}$ will be used for the set of all m×n real matrices. Given a matrix *A*, its transpose and inverse will be denoted as $A^{T}$ and $A^{-1}$, respectively. $I_{n}$ and $1_{n}$ will denote the n×n identity matrix and the n×1 all-ones vector, respectively. The dimensions of vectors and matrices, when not explicitly stated, will be assumed to be compatible with algebraic operations; for instance, *I* and 1 will denote the identity matrix and the all-ones vector of appropriate dimensions, respectively. The shorthand $\left(M_{1}|\cdots |M_{l}\right)$ will represent a partitioned matrix whose blocks are the submatrices $M_{1},\ldots ,M_{l}$. The symbols ⊗ and ∘ will be used to denote the Kronecker and Hadamard product of matrices, respectively, and $\delta_{k,l}$ will represent the Kronecker delta function. If *a* and *b* are arbitrary random vectors, we will use the notation $\bar{a}=E\left[a\right]$ for the mean vector, where E[·] is the mathematical expectation operator and $Cov\left[a,b\right]=E\left[\left(a-\bar{a}\right){\left(b-\bar{b}\right)}^{T}\right]=E\left[ab^{T}\right]-\bar{a}{\bar{b}}^{T}$ for the covariance matrix (if a=b, we will just write Cov[a]). Given a function $\Gamma_{k,l}$, depending on the time instants *k* and *l*, we will simply write $\Gamma_{k}$ when k=l; similarly, for any function $\Xi^{\left(rs\right)}$, depending on the sensor nodes *r* and *s*, $\Xi^{\left(r\right)}$ will be written when r=s. Finally, the abbreviations LS and MSE will hold for least-squares and the mean squared error, respectively.

## 2. System Description and Preliminaries

We are concerned with the estimation problem over a sensor network constituted by a group of spatially distributed sensor nodes that connect to each other according to a fixed network topology, represented by a directed graph, subject to random uncertainties including fading measurements and white multiplicative and additive noises. Every node in the network takes measurements that are linearly related to the unobserved system state and are susceptible to suffering deception attacks, causing, at each sampling time, the measurements available for the estimation at each sensor to be able to be randomly either the actual measurement or just the noise injected by the attacker. Our aim is to design a distributed filtering algorithm to estimate, for every node, the state of the system at a particular time, based on the local measurements and neighboring information up to that time.

The system model and the hypotheses about the processes involved in it are described in the following paragraphs.

### 2.1. Stochastic Uncertain System Model

Consider a discrete-time stochastic uncertain networked system with uncertainties caused by white additive and multiplicative noises in the state and observation equations. More precisely, the state evolution, including perturbations described by multiplicative and additive noises, is described by the following time-varying equation:

(1) $$x_{k+1}=\left(F_{k}+\alpha_{k}{\overset{˘}{F}}_{k}\right)x_{k}+w_{k},\;k\geq 0,$$

where $x_{k}\in R^{n_{x}}$ is the state vector at time k≥0, which cannot be directly observed, ${\left\{w_{k}\right\}}_{k\geq 0}$ is the white process noise, ${\left\{\alpha_{k}\right\}}_{k\geq 0}$ is a scalar white multiplicative noise, and $F_{k}$, ${\overset{˘}{F}}_{k}$ are known time-dependent matrices.

The sensor network is composed of *m* sensor nodes, and the state measured outputs from the different sensor nodes are described by:

(2) $${\overset{˘}{y}}_{k}^{\left(i\right)}=\theta_{k}^{\left(i\right)}\left(H_{k}^{\left(i\right)}+\beta_{k}^{\left(i\right)}{\overset{˘}{H}}_{k}^{\left(i\right)}\right)x_{k}+v_{k}^{\left(i\right)},\;\;\;\;k\geq 1,\;\;\;\;\;i=1,\ldots ,m,$$

where, for i=1,…,m, ${\overset{˘}{y}}_{k}^{\left(i\right)}\in R^{n_{y}}$ is the measurement output from sensor node *i*, ${\left\{v_{k}^{\left(i\right)}\right\}}_{k\geq 1}$ are the additive white measurement noises, ${\left\{\theta_{k}^{\left(i\right)}\right\}}_{k\geq 1}$ are [0,1]-valued random variables, ${\left\{\beta_{k}^{\left(i\right)}\right\}}_{k\geq 1}$ are the scalar white multiplicative noises, and ${\left\{H_{k}^{\left(i\right)}\right\}}_{k\geq 1}$, ${\left\{{\overset{˘}{H}}_{k}^{\left(i\right)}\right\}}_{k\geq 1}$ are known time-varying matrices.

The following assumptions about the initial state and the additive and multiplicative noises involved in the multi-sensor system model (1)–(2) are required to address the estimation problem at hand:

(i) The initial state $x_{0}$ is a random vector with zero mean and $Cov\left[x_{0}\right]=\Sigma_{0}^{x}$.
(ii) ${\left\{\theta_{k}^{\left(i\right)}\right\}}_{k\geq 1},\;i=1,\ldots ,m$ are sequences of independent random variables with an arbitrary distribution over the interval [0,1], with known means and variances; we will denote them as $E\left[\theta_{k}^{\left(i\right)}\right]={\bar{\theta }}_{k}^{\left(i\right)}$ and $Var\left(\theta_{k}^{\left(i\right)}\right)=V_{k}^{\theta^{\left(i\right)}}$.
(iii) ${\left\{\alpha_{k}\right\}}_{k\geq 0}$ and ${\left\{\beta_{k}^{\left(i\right)}\right\}}_{k\geq 1}$, i=1,…,m, are scalar white processes with zero means and known variances, $Var\left(\alpha_{k}\right)=V_{k}^{\alpha }$ and $Var\left(\beta_{k}^{\left(i\right)}\right)=V_{k}^{\beta^{\left(i\right)}}$.
(iv) The noises ${\left\{w_{k}\right\}}_{k\geq 0}$ and ${\left\{v_{k}^{\left(i\right)}\right\}}_{k\geq 1}$, i=1,…,m, are zero-mean second-order white processes with known covariance and cross-covariance matrices:
  $$Cov\left[w_{k},w_{l}\right]=Q_{k}\delta_{k,l},\;\;\;\;Cov\left[v_{k}^{\left(i\right)},v_{l}^{\left(j\right)}\right]=R_{k}^{\left(ij\right)}\delta_{k,l},\;\;\;\;Cov\left[w_{k},v_{l}^{\left(i\right)}\right]=S_{k,k+1}^{\left(i\right)}\delta_{k+1,l},\;\;\;\;\;i,j=1,\ldots ,m.$$
(v) The initial state $x_{0}$ and the processes ${\left\{\alpha_{k}\right\}}_{k\geq 0}$, ${\left\{\beta_{k}^{\left(i\right)}\right\}}_{k\geq 1}$ and ${\left\{\theta_{k}^{\left(i\right)}\right\}}_{k\geq 1},\;i=1,\ldots ,m$, are mutually independent, and they are independent of the additive noises ${\left\{w_{k}\right\}}_{k\geq 0}$ and ${\left\{v_{k}^{\left(i\right)}\right\}}_{k\geq 1},\;i=1,\ldots ,m.$

**Remark** **1.** *From the above assumptions, it is easy to see that the state covariance function can be expressed as $Cov\left[x_{k},x_{l}\right]=F_{k,l}\Sigma_{l}^{x},\;l<k,$ where $F_{k,l}=F_{k-1}\cdots F_{l}$, and the matrices $\Sigma_{l}^{x}\equiv Cov\left[x_{l}\right],\;l\geq 1,$ are recursively obtained by:*

$$\Sigma_{l}^{x}=F_{l-1}\Sigma_{l-1}^{x}F_{l-1}^{T}+V_{l-1}^{\alpha }{\overset{˘}{F}}_{l-1}\Sigma_{l-1}^{x}{\overset{˘}{F}}_{l-1}^{T}+Q_{l-1},\;\;\;\;l\geq 1.$$

*Hence, denoting $F_{k}=F_{k,0}$ and $B_{l}^{T}=F_{l,0}^{-1}\Sigma_{l}^{x}$, the state covariance function can be expressed in a separable form; namely,*

(3) $$Cov\left[x_{k},x_{l}\right]=F_{k}B_{l}^{T},\;\;\;\;l\leq k.$$

*Moreover, for i=1,…,m, since the sensor noises at each instant are correlated with the process noise at the previous time (Assumption (iii)), we have that $Cov\left[x_{k},v_{l}^{\left(i\right)}\right]=F_{k,l}E\left[x_{l}v_{l}^{(i)T}\right]=F_{k,l}E\left[w_{l-1}v_{l}^{(i)T}\right]=F_{k,l}S_{l-1,l}^{\left(i\right)}$, for l<k, $Cov\left[x_{k},v_{k}^{\left(i\right)}\right]=S_{k-1,k}^{\left(i\right)}$, and $Cov[x_{k},v_{l}^{\left(i\right)}]=0$, for l>k. Hence, each sensor noise is correlated with the state at the same and subsequent time steps, and denoting $C_{l}^{(i)T}=F_{l,0}^{-1}S_{l-1,l}^{\left(i\right)}$, i=1,…,m, this correlation can also be expressed in a separable form as follows:*

(4) $$Cov\left[x_{k},v_{l}^{\left(i\right)}\right]=\left\{\begin{array}{cc} F_{k}C_{l}^{(i)T}, & l\leq k,\\ 0, & l>k. \end{array}\right.$$

### 2.2. Network Topology and Deception Attacks Model

We consider that the spatial distribution of the sensor nodes corresponds to a fixed network topology, represented by a digraph G=(V,E,A) of order *m*, where $V=\left\{1,\ldots ,m\right\}$ is the set of sensor nodes, E⊆V×V is the set of edges, and $A={\left(a_{ij}\right)}_{m\times m}$ is the adjacency matrix, with binary entries $a_{ij}$ indicating the link relation among sensors; namely, for i≠j, $a_{ij}=1$ if (i,j)∈E, meaning that sensor *i* receives information from sensor *j*, and $a_{ij}=0$ otherwise. Since any sensor gets its own information, it is assumed that $a_{ii}=1,\;\forall i\in V$. The neighborhood of node *i* is defined as the set of adjacent nodes plus the node itself and will be denoted by $N_{i}=\left\{j\in V:\;a_{ij}=1\right\},\;\forall i\in V$; it is assumed that each node *i* knows all the relevant information from its neighboring nodes $j\in N_{i}$.

In this paper, it is considered that deception attacks are launched by potential adversaries at the sensor measured outputs, injecting false information that will randomly perturb the real measurements. Specifically, at the *i*th sensor, i=1,…,m, the deception signal $\xi_{k}^{\left(i\right)}$ is modeled as a two-component sum:

(5) $$\xi_{k}^{\left(i\right)}=-{\overset{˘}{y}}_{k}^{\left(i\right)}+\varepsilon_{k}^{\left(i\right)},\;\;\;k\geq 1;\;\;\;\;\;i=1,\ldots ,m.$$

The first component, $-{\overset{˘}{y}}_{k}^{\left(i\right)}$, neutralizes the actual sensor measurements, and the second one, $\varepsilon_{k}^{\left(i\right)}$, is a noise that represents the deceptive information added by the adversary. The following assumption is required on the noises ${\left\{\varepsilon_{k}^{\left(i\right)}\right\}}_{k\geq 1}$, i=1,…,m:

(vi) The noises ${\left\{\varepsilon_{k}^{\left(i\right)}\right\}}_{k\geq 1}$i=1,…,m, are independent zero-mean second-order white processes with known covariance matrices:
  $$Cov\left[\varepsilon_{k}^{\left(i\right)},\varepsilon_{l}^{\left(j\right)}\right]=T_{k}^{\left(ij\right)}\delta_{k,l},\;\;\;k,l\geq 1;\;\;\;\;\;i,j=1,\ldots ,m.$$
  Moreover, they are independent of the other noise processes involved in the system.

Assuming that the attacks launched by the adversaries may randomly succeed or not, the *i*th sensor measurements, $y_{k}^{\left(i\right)}$, subject to the deception attacks are modeled by:

(6) $$y_{k}^{\left(i\right)}={\overset{˘}{y}}_{k}^{\left(i\right)}+\lambda_{k}^{\left(i\right)}\xi_{k}^{\left(i\right)},\;\;\;k\geq 1;\;\;\;\;\;i=1,\ldots ,m,$$

where ${\left\{\lambda_{k}^{\left(i\right)}\right\}}_{k\geq 1}$, i=1,…,m, are different white sequences of Bernoulli random variables introduced to model whether the deception attacks actually happen or not. In fact, the value $\lambda_{k}^{\left(i\right)}=1$ models a successful attack on the *i*th sensor at time *k*, meaning that only noise $\varepsilon_{k}^{\left(i\right)}$ is used for the estimation, while the value $\lambda_{k}^{\left(i\right)}=0$ models a failed attack, which means that the real measured output ${\overset{˘}{y}}_{k}^{\left(i\right)}$ is used.

The following assumption is imposed on these Bernoulli random variables:

(vii) ${\left\{\lambda_{k}^{\left(i\right)}\right\}}_{k\geq 1}$, i=1,…,m, are independent white sequences of Bernoulli random variables with known probabilities:
  $$P\left(\lambda_{k}^{\left(i\right)}=1\right)={\bar{\lambda }}_{k}^{\left(i\right)},\;\;\;k\geq 1;\;\;\;\;\;i=1,\ldots ,m.$$
  Furthermore, these sequences are independent of the other noise processes involved in the system.

## 3. Problem Formulation and Tools

### 3.1. Distributed Filtering Problem and Design Phases of the Algorithm

Our aim is to design, at each sensor node, a distributed filtering technique over the networked uncertain system with fading measurements subject to deception attacks, described by Equations (1), (2), (5), and (6), under Assumptions (i)–(vii). As described in the previous section, the sensor nodes are linked according to a fixed network topology; the communication between neighboring nodes is assumed to obey the following scheme: first, every node receives the measurements from its adjacent nodes, and after that, it receives all the estimators obtained by its adjacent nodes from the measurements previously received.

In order to make the most out of this communication scheme among neighboring nodes, the distributed filter proposed in this paper will be obtained in two phases:

Phase 1: For i=1,…,m, the least-squares (LS) linear filter of the state $x_{k}$ will be recursively obtained at the *i*th sensor node, using its own measured outputs and those provided by its neighboring nodes (all randomly affected by deception attacks). These estimators, denoted by ${\hat{x}}_{k/k}^{\left(i\right)}$, k≥0, i=1,…,m, will be called intermediate filters.

In order to design these intermediate filters, we need to select the measurements available at the *i*th sensor node at the time instant *k*, $\left\{y_{1}^{\left(j\right)},\ldots ,y_{k}^{\left(j\right)};\;j\in N_{i}\right\}$. For this purpose, we consider the $mn_{y}\times 1$ vector obtained by gathering all available measurements from the different sensors at the sampling time *l*, $y_{l}={\left(y_{l}^{(1)T},\ldots ,y_{l}^{(m)T}\right)}^{T}$, l=1,…,k, and the matrix $A_{y}^{\left(i\right)}$ obtained by removing the all-zero rows of:

$$Diag\left(a_{i1},\ldots ,a_{im}\right)⊗I_{n_{y}}=\left(\begin{array}{ccc} a_{i1}I_{n_{y}} & \cdots & 0\\ \vdots & \ddots & \vdots \\ 0 & \cdots & a_{im}I_{n_{y}} \end{array}\right),\;i=1,\ldots ,m.$$

Since the (i,j) entry of the adjacency matrix, $a_{ij}$, is equal to one if, and only if, $j\in N_{i}$ (meaning that node *i* can receive information from node *j*), the product:

(7) $$Y_{l}^{\left(i\right)}=A_{y}^{\left(i\right)}y_{l},\;\;l=1,\ldots ,k,$$

yields the vector constituted by the measured outputs of the *i*th sensor and those provided by its adjacent nodes.

Then, our aim in the first phase is to derive a recursive algorithm to obtain, for each sensor node *i*, the LS linear estimator of the state $x_{k}$ based on the measurement vectors $\left\{Y_{1}^{\left(i\right)},\ldots ,Y_{k}^{\left(i\right)}\right\}$.

Phase 2: The fact that every node can share its own intermediate estimator within its communication neighborhood brings about the possibility of fusing, at each sensor node *i*, its own intermediate filter and the intermediate filters from its adjacent nodes. More precisely, at the *i*th sensor node, the proposed distributed filter, ${\hat{x}}_{k/k}^{d(i)}$, will be generated as the optimal (minimum mean squared error) matrix-weighted linear combination of the intermediate estimators, ${\hat{x}}_{k/k}^{\left(j\right)}$, obtained in its neighboring nodes $j\in N_{i}$.

Analogous to the previous phase, denoting now ${\hat{X}}_{k/k}={\left({\hat{x}}_{k/k}^{(1)T},\ldots ,{\hat{x}}_{k/k}^{(m)T}\right)}^{T}$ the $mn_{x}\times 1$ vector obtained by gathering all the intermediate estimators of the different sensors, we can select those received by the *i*th sensor node, $\left\{{\hat{x}}_{k/k}^{\left(j\right)};\;j\in N_{i}\right\}$, by considering the product:

(8) $${\hat{X}}_{k/k}^{\left(i\right)}=A_{x}^{\left(i\right)}{\hat{X}}_{k/k},$$

where $A_{x}^{\left(i\right)}$ is the matrix obtained by removing the all-zeros rows of:

$$Diag\left(a_{i1},\ldots ,a_{im}\right)⊗I_{n_{x}}=\left(\begin{array}{ccc} a_{i1}I_{n_{x}} & \cdots & 0\\ \vdots & \ddots & \vdots \\ 0 & \cdots & a_{im}I_{n_{x}} \end{array}\right),\;i=1,\ldots ,m.$$

Hence, our aim in this second phase is to find the optimal weighting matrices $G_{k}^{\left(i\right)}$, minimizing the mean squared error (MSE):

(9) $$E\left[{\left(x_{k}-G_{k}^{\left(i\right)}{\hat{X}}_{k/k}^{\left(i\right)}\right)}^{T}\left(x_{k}-G_{k}^{\left(i\right)}{\hat{X}}_{k/k}^{\left(i\right)}\right)\right].$$

### 3.2. Gathered Measurement Model and Properties

According to the previous considerations, in order to address the intermediate filtering problem at every sensor node, we need to deal with the gathered measurement vector, $y_{k}={\left(y_{k}^{(1)T},\ldots ,y_{k}^{(m)T}\right)}^{T}$, obtained by stacking the measured outputs provided by all sensors at the sampling time k≥1, from which we must extract the measurements received from the sensor neighbors. Before moving on to the actual design of the intermediate filters, we will establish the mathematical model that describes the relationship between these gathered measurements and the system state, as well as the statistical properties of the processes involved in this model.

Hereafter, the following notation will be used:

$${\overset{˘}{y}}_{k}=\left(\begin{array}{c} {\overset{˘}{y}}_{k}^{\left(1\right)}\\ \vdots \\ {\overset{ˇ}{y}}_{k}^{\left(m\right)} \end{array}\right),\;H_{k}=\left(\begin{array}{c} H_{k}^{\left(1\right)}\\ \vdots \\ H_{k}^{\left(m\right)} \end{array}\right),\;{\overset{˘}{H}}_{k}=\left(\begin{array}{c} {\overset{˘}{H}}_{k}^{\left(1\right)}\\ \vdots \\ {\overset{˘}{H}}_{k}^{\left(m\right)} \end{array}\right),\;v_{k}=\left(\begin{array}{c} v_{k}^{\left(1\right)}\\ \vdots \\ v_{k}^{\left(m\right)} \end{array}\right),\;$$

and for any m×1 vector $b_{k}={\left(b_{k}^{\left(1\right)},\ldots ,b_{k}^{\left(m\right)}\right)}^{T}$, we will denote:

$$D_{k}^{b}=\left(\begin{array}{ccc} b_{k}^{\left(1\right)} & \cdots & 0\\ \vdots & \ddots & \vdots \\ 0 & \cdots & b_{k}^{\left(m\right)} \end{array}\right)⊗I_{n_{y}}.$$

From (2), the vectors ${\left\{{\overset{˘}{y}}_{k}\right\}}_{k\geq 1}$ clearly satisfy:

(10) $${\overset{˘}{y}}_{k}=D_{k}^{\theta }\left(H_{k}+D_{k}^{\beta }{\overset{˘}{H}}_{k}\right)x_{k}+v_{k},\;\;k\geq 1,$$

and the following properties of the processes involved in this gathered observation equation are easily derived from Assumptions (i)–(v).

**Proposition** **1.**

*(a)*  *The observation matrices in (10), $D_{k}^{\theta }\left(H_{k}+D_{k}^{\beta }{\overset{˘}{H}}_{k}\right)$, satisfy:*
  · $E\left[D_{k}^{\theta }\left(H_{k}+D_{k}^{\beta }{\overset{˘}{H}}_{k}\right)\right]=D_{k}^{\bar{\theta }}H_{k}$
  · *The (i,j) entry of $E\left[D_{k}^{\theta }\left(H_{k}+D_{k}^{\beta }{\overset{˘}{H}}_{k}\right)\Sigma_{k}^{x}{\left(H_{k}+D_{k}^{\beta }{\overset{˘}{H}}_{k}\right)}^{T}\;\;D_{k}^{\theta }\right],\;i,j=1,\ldots ,m,$ is given by:*
    $$\begin{array}{c} E\left[\theta_{k}^{\left(i\right)}\left(H_{k}^{\left(i\right)}+\beta_{k}^{\left(i\right)}{\overset{˘}{H}}_{k}^{\left(i\right)}\right)\Sigma_{k}^{x}\theta_{k}^{\left(j\right)}{\left(H_{k}^{\left(j\right)}+\beta_{k}^{\left(j\right)}{\overset{˘}{H}}_{k}^{\left(j\right)}\right)}^{T}\right]=\\ \;=\left\{\begin{array}{cc} {\bar{\theta }}_{k}^{\left(i\right)}{\bar{\theta }}_{k}^{\left(j\right)}H_{k}^{\left(i\right)}\Sigma_{k}^{x}H_{k}^{(j)T}, & i\neq j,\\ \left(V_{k}^{\theta^{\left(i\right)}}+{\left({\bar{\theta }}_{k}^{\left(i\right)}\right)}^{2}\right)H_{k}^{\left(i\right)}\Sigma_{k}^{x}H_{k}^{(i)T}+V_{k}^{\beta^{\left(i\right)}}{\overset{˘}{H}}_{k}^{\left(i\right)}\Sigma_{k}^{x}{\overset{˘}{H}}_{k}^{(i)T}, & i=j. \end{array}\right. \end{array}$$
*(b)*  *The noise ${\left\{v_{k}\right\}}_{k\geq 1}$ is a zero-mean white process with $Cov\left[v_{k}\right]=R_{k}={\left(R_{k}^{\left(ij\right)}\right)}_{i,j=1,\ldots ,m}$.*
*Moreover, the noise process ${\left\{v_{k}\right\}}_{k\geq 1}$ is correlated with the state process ${\left\{x_{k}\right\}}_{k\geq 1}$ at the same and subsequent time steps; namely, from (4), denoting $C_{l}^{T}=\left(C_{l}^{\left(1\right)}\left|\cdots \right|C_{k}^{\left(m\right)}\right),$ we have that:*
  (11) $$E\left[x_{k}v_{l}^{T}\right]=\left\{\begin{array}{cc} F_{k}C_{l}^{T}, & l\leq k,\\ 0, & l>k. \end{array}\right.$$
*Note that, for l=k, we have $E\left[x_{k}v_{k}^{T}\right]=F_{k}C_{k}^{T}=S_{k-1,k}$, with $S_{k-1,k}=\left(S_{k-1,k}^{\left(1\right)}\left|\cdots \right|S_{k-1,k}^{\left(m\right)}\right).$*

*Next, using (5) and (6), the following equation for the gathered measurement vectors ${\left\{y_{k}\right\}}_{k\geq 1}$ is deduced:*

(12) $$y_{k}=\left(I-D_{k}^{\lambda }\right){\overset{˘}{y}}_{k}+D_{k}^{\lambda }\varepsilon_{k},\;\;k\geq 1,$$

*where $\varepsilon_{k}={\left(\varepsilon_{k}^{(1)T},\ldots ,\varepsilon_{k}^{(m)T}\right)}^{T}$, and the following properties hold true:*

**Proposition** **2.**

*(a)*  *${\left\{D_{k}^{\lambda }\right\}}_{k\geq 1}$ is a sequence of independent random matrices with $E\left[D_{k}^{\lambda }\right]=D_{k}^{\bar{\lambda }}$, k≥1, and denoting $\lambda_{k}={\left(\lambda_{k}^{\left(1\right)}1_{n_{y}}^{T},\ldots ,\lambda_{k}^{\left(m\right)}1_{n_{y}}^{T}\right)}^{T}={\left(\lambda_{k}^{\left(1\right)},\ldots ,\lambda_{k}^{\left(m\right)}\right)}^{T}⊗1_{n_{y}}$, k≥1, the matrices $K_{k}^{\lambda }\equiv E\left[\lambda_{k}\lambda_{k}^{T}\right]$ and $K_{k}^{1-\lambda }\equiv E\left[\left(1-\lambda_{k}\right){\left(1-\lambda_{k}\right)}^{T}\right]$ are known, while their entries can be calculated taking into account that, from Assumption (vii), $E\left[\lambda_{k}^{\left(i\right)}\lambda_{k}^{\left(j\right)}\right]=\left\{\begin{array}{c} {\bar{\lambda }}_{k}^{\left(i\right)},\;\;i=j,\;\\ {\bar{\lambda }}_{k}^{\left(i\right)}{\bar{\lambda }}_{k}^{\left(j\right)},\;\;i\neq j. \end{array}\right.$*
*(b)*  *The noise ${\left\{\varepsilon_{k}\right\}}_{k\geq 1}$ is a zero-mean white process with $Cov\left[\varepsilon_{k}\right]=T_{k}={\left(T_{k}^{\left(ij\right)}\right)}_{i,j=1,\ldots ,m}$.*

**Remark** **2.** *From the above properties, it is easy to show that ${\left\{{\overset{˘}{y}}_{k}\right\}}_{k\geq 1}$ is a zero-mean second-order process, and the covariance matrices $\Sigma_{k}^{y}\equiv Cov\left[y_{k}\right]$ verify:*

(13) $$\Sigma_{k}^{y}=K_{k}^{1-\lambda }\circ \Sigma_{k}^{\overset{˘}{y}}+K_{k}^{\lambda }\circ T_{k},\;\;k\geq 1,$$

*where $\Sigma_{k}^{\overset{˘}{y}}\equiv Cov\left[{\overset{˘}{y}}_{k}\right]$ are obtained by:*

(14) $$\Sigma_{k}^{\overset{˘}{y}}=E\left[D_{k}^{\theta }\left(H_{k}+D_{k}^{\beta }{\overset{˘}{H}}_{k}\right)\Sigma_{k}^{x}{\left(H_{k}+D_{k}^{\beta }{\overset{˘}{H}}_{k}\right)}^{T}D_{k}^{\theta }\right]+R_{k}+D_{k}^{\bar{\theta }}\left(H_{k}S_{k-1,k}+S_{k-1,k}^{T}H_{k}^{T}\right),\;\;k\geq 1.$$

## 4. Main Results

We are now in a position to address the state distributed filtering problem for the networked uncertain system with fading measurements subject to deception attacks described in Section 2, which, according to the considerations made in Section 3, is reduced to the problem of obtaining, at each sensor *i*, i=1,…,m, the LS linear estimator of the state $x_{k}$ based on the measurement vectors $\left\{Y_{1}^{\left(i\right)},\ldots ,Y_{k}^{\left(i\right)}\right\}$ (intermediate filter at the *i*th sensor, ${\hat{x}}_{k/k}^{\left(i\right)}$) and then fusing the resulting estimator with those obtained at its adjacent nodes, to yield the minimum MSE matrix-weighted linear combination (distributed filter at the *i*th sensor, ${\hat{x}}_{k/k}^{d(i)}$).

It must be indicated that, although the most popular approaches to obtain recursive estimation algorithms when the state evolution equation is known are based on the state-space model, the filtering algorithms proposed in this section will be obtained without requiring the transition equation for the state vector (1). Only the factorization of the state covariance matrix in a separable form (3) will be used in the mathematical derivations of the filtering algorithms, and therefore, such algorithms are widely useful to estimate stationary or non-stationary state processes whose covariance matrix admits this kind of factorization. As a consequence of this approach, the proposed algorithm structure is different from that of most standard algorithms based on the state-space model, although it can be reformulated as a prediction-update algorithm, similar to the conventional ones (see Remark 3).

### 4.1. Recursive Intermediate Filtering Algorithm

Let us start by developing, at every sensor node *i*, a recursive algorithm for the intermediate filtering estimator, ${\hat{x}}_{k/k}^{\left(i\right)}$; that is, the LS linear estimator of the state $x_{k}$ based on the measurement vectors $\left\{Y_{1}^{\left(i\right)},\ldots ,Y_{k}^{\left(i\right)}\right\}$. The following theorem provides the design procedure for such estimators and their error covariance matrices.

**Theorem** **1.** *For the ith sensor, i=1,…,m, the LS linear filtering estimator ${\hat{x}}_{k/k}^{\left(i\right)}$ and the error covariance matrices, $P_{k/k}^{\left(i\right)}\equiv Cov\left[x_{k}-{\hat{x}}_{k/k}^{\left(i\right)}\right]$, are obtained from the following recursive algorithm:*

(15) $${\hat{x}}_{k/k}^{\left(i\right)}=F_{k}u_{k}^{\left(i\right)},\;\;k\geq 1,$$

(16) $$P_{k/k}^{\left(i\right)}=\Sigma_{k}^{x}-F_{k}\Sigma_{k}^{u^{\left(i\right)}}F_{k}^{T},\;\;k\geq 1,$$

*where $F_{k}=\left(F_{k}|F_{k}\right)$, and the vectors $u_{k}^{\left(i\right)}$ satisfy the following recursive relation:*

(17) $$u_{k}^{\left(i\right)}=u_{k-1}^{\left(i\right)}+U_{k}^{\left(i\right)}\Pi_{k}^{(i)-1}\mu_{k}^{\left(i\right)},\;\;k\geq 1;\;\;\;\;\;\;\;u_{0}^{\left(i\right)}=0.$$

*The matrices $\Sigma_{k}^{x}=Cov\left[x_{k}\right]$ are given in (3), and $\Sigma_{k}^{u^{\left(i\right)}}\equiv Cov\left[u_{k}^{\left(i\right)}\right]$ are recursively calculated by:*

(18) $$\Sigma_{k}^{u^{\left(i\right)}}=\Sigma_{k-1}^{u^{\left(i\right)}}+U_{k}^{\left(i\right)}\Pi_{k}^{(i)-1}U_{k}^{(i)T},\;\;k\geq 1;\;\;\;\;\;\;\;\Sigma_{0}^{u^{\left(i\right)}}=0,$$

*where:*

(19) $$U_{k}^{\left(i\right)}=\left({\left(D_{k}^{\bar{\theta }}H_{k}B_{k}|C_{k}\right)}^{T}-\Sigma_{k-1}^{u^{\left(i\right)}}F_{k}^{T}H_{k}^{T}D_{k}^{\bar{\theta }}\right)\left(I-D_{k}^{\bar{\lambda }}\right)A_{y}^{(i)T},\;\;k\geq 1.$$

*The vectors $\mu_{k}^{\left(i\right)}$ and their covariance matrices $\Pi_{k}^{\left(i\right)}\equiv Cov\left[\mu_{k}^{\left(i\right)}\right]$ satisfy:*

(20) $$\mu_{k}^{\left(i\right)}=A_{y}^{\left(i\right)}\left(y_{k}-\left(I-D_{k}^{\bar{\lambda }}\right)D_{k}^{\bar{\theta }}H_{k}F_{k}u_{k-1}^{\left(i\right)}\right),\;\;k\geq 1,$$

(21) $$\Pi_{k}^{\left(i\right)}=A_{y}^{\left(i\right)}\left(\Sigma_{k}^{y}-\left(I-D_{k}^{\bar{\lambda }}\right)D_{k}^{\bar{\theta }}H_{k}F_{k}\Sigma_{k-1}^{u^{\left(i\right)}}F_{k}^{T}H_{k}^{T}D_{k}^{\bar{\theta }}\left(I-D_{k}^{\bar{\lambda }}\right)\right)A_{y}^{(i)T},\;\;k\geq 1,$$

*where $\Sigma_{k}^{y}=Cov\left[y_{k}\right]$ is calculated from (13).*

**Proof.**  The design of the intermediate estimators is based on an innovation approach, according to which the LS linear estimator of the state $x_{k}$ based on the measurement vectors $\left\{Y_{1}^{\left(i\right)},\ldots ,Y_{k}^{\left(i\right)}\right\}$ admits the following expression as a linear combination of the innovations $\mu_{l}^{\left(i\right)}=Y_{l}^{\left(i\right)}-{\hat{Y}}_{l/l-1}^{\left(i\right)},\;l=1,\ldots ,k$:

$${\hat{x}}_{k/k}^{\left(i\right)}=\sum_{l=1}^{k}X_{k,l}^{\left(i\right)}\Pi_{l}^{(i)-1}\mu_{l}^{\left(i\right)},\;\;i=1,\ldots ,m,$$

where $X_{k,l}^{\left(i\right)}=Cov\left[x_{k},\mu_{l}^{\left(i\right)}\right]$ and $\Pi_{l}^{\left(i\right)}=Cov\left[\mu_{l}^{\left(i\right)}\right]$.

In order to obtain these covariance functions, let us observe that, from (7), the innovations can be written as $\mu_{k}^{\left(i\right)}=A_{y}^{\left(i\right)}\left(y_{k}-{\hat{y}}_{k/k-1}^{\left(i\right)}\right)$, where ${\hat{y}}_{k/k-1}^{\left(i\right)}$ is the LS linear estimator of $y_{k}$ based on $\left\{Y_{1}^{\left(i\right)},\ldots ,Y_{k-1}^{\left(i\right)}\right\}$. Hence,

$$X_{k,l}^{\left(i\right)}=\left(E\left[x_{k}y_{l}^{T}\right]-E\left[x_{k}{\hat{y}}_{l/l-1}^{(i)T}\right]\right)A_{y}^{(i)T},\;\;\;\;1\leq l\leq k.$$

Using (10) and (12) and taking into account the model assumptions, the observation predictors ${\hat{y}}_{l/l-1}^{\left(i\right)}$ are expressed as:

(22) $${\hat{y}}_{l/l-1}^{\left(i\right)}=\left(I-D_{l}^{\bar{\lambda }}\right)D_{l}^{\bar{\theta }}H_{l}{\hat{x}}_{l/l-1}^{\left(i\right)},\;\;\;l\geq 1.$$

This, together with (12) and the covariance factorizations (3) and (11), implies that the following equalities are true for l=1,…,k:

$$\begin{array}{c} E\left[x_{k}y_{l}^{T}\right]=\left(F_{k}B_{l}^{T}H_{l}^{T}D_{l}^{\bar{\theta }}+F_{k}C_{l}^{T}\right)\left(I-D_{l}^{\bar{\lambda }}\right)\\ E\left[x_{k}{\hat{y}}_{l/l-1}^{(i)T}\right]=E\left[x_{k}{\hat{x}}_{l/l-1}^{(i)T}\right]H_{l}^{T}D_{l}^{\bar{\theta }}\left(I-D_{l}^{\bar{\lambda }}\right). \end{array}$$

Expressing now the one-step predictor, ${\hat{x}}_{l/l-1}^{\left(i\right)}$, as a linear combination of the innovations:

$${\hat{x}}_{l/l-1}^{\left(i\right)}=\sum_{h=1}^{l-1}X_{l,h}^{\left(i\right)}\Pi_{j}^{(i)-1}\mu_{h}^{\left(i\right)},\;\;l\geq 2;\;\;\;\;x_{1/0}^{\left(i\right)}=0,$$

and denoting $F_{k}=\left(F_{k}|F_{k}\right)$, we conclude that:

$$X_{k,l}^{\left(i\right)}=F_{k}{\left(D_{k}^{\bar{\theta }}H_{l}B_{l}|C_{l}\right)}^{T}-\left(1-\delta_{1,l}\right)\sum_{h=1}^{l-1}X_{k,h}^{\left(i\right)}\Pi_{h}^{\left(i\right)}X_{l,h}^{(i)T}H_{l}^{T}D_{l}^{\bar{\theta }})\left(I-D_{l}^{\bar{\lambda }}\right)A_{y}^{(i)T},\;\;\;1\leq l\leq k.$$

This identity guarantees that $X_{k,l}^{\left(i\right)}$ can be factorized as $X_{k,l}^{\left(i\right)}=F_{k}U_{l}^{\left(i\right)},\;\;1\leq l\leq k$, with:

(23) $$U_{l}^{\left(i\right)}=\left({\left(D_{k}^{\bar{\theta }}H_{l}B_{l}|C_{l}\right)}^{T}-\left(1-\delta_{l,1}\right)\sum_{h=1}^{l-1}U_{h}^{\left(i\right)}\Pi_{h}^{(i)-1}U_{h}^{(i)T}F_{l}^{T}H_{l}^{T}D_{l}^{\bar{\theta }}\right)\left(I-D_{l}^{\bar{\lambda }}\right)A_{y}^{(i)T},\;\;\;1\leq l\leq k.$$

Define the vectors:

(24) $$u_{k}^{\left(i\right)}\equiv \sum_{h=1}^{k}U_{h}^{\left(i\right)}\Pi_{h}^{(i)-1}\mu_{h}^{\left(i\right)},\;\;k\geq 1;\;\;\;\;\;u_{0}^{\left(i\right)}\equiv 0,$$

and note that their covariances, $\Sigma_{k}^{u^{\left(i\right)}}=Cov\left[u_{k}^{\left(i\right)}\right]$, satisfy:

(25) $$\Sigma_{k}^{u^{\left(i\right)}}=\sum_{h=1}^{k}U_{h}^{\left(i\right)}\Pi_{h}^{(i)-1}U_{h}^{(i)T},\;\;k\geq 1;\;\;\;\;\;\;\;\Sigma_{0}^{u^{\left(i\right)}}=0,$$

Expression (15) for the filtering estimator is immediately derived, and also, it is clear that the state predictor is given by ${\hat{x}}_{k/k-1}^{\left(i\right)}=F_{k}u_{k-1}^{\left(i\right)},\;k\geq 1.$ From (15), Equation (16) for the error covariance matrices is also easily proven just taking into account that, since the estimation error is uncorrelated with the estimator, $P_{k/k}^{\left(i\right)}=Cov\left[x_{k}\right]-Cov\left[{\hat{x}}_{k/k}^{\left(i\right)}\right]$. The recursive relations (17) and (18) are immediately derived from (24) and (25), respectively, and Expression (19) is straightforward from (23) and (25).

From (22), the innovation at time *k* is given by:

(26) $$\mu_{k}^{\left(i\right)}=A_{y}^{\left(i\right)}\left(y_{k}-\left(I-D_{k}^{\bar{\lambda }}\right)D_{k}^{\bar{\theta }}H_{k}{\hat{x}}_{k/k-1}^{\left(i\right)}\right),\;\;k\geq 1,$$

which leads to Expression (20) just taking into consideration that ${\hat{x}}_{k/k-1}^{\left(i\right)}=F_{k}u_{k-1}^{\left(i\right)}.$

Finally, to obtain Expression (21) for the innovation covariance matrices, $\Pi_{k}^{\left(i\right)}=Cov\left[\mu_{k}^{\left(i\right)}\right]$, we use again that the estimation error and the estimator are uncorrelated, which guarantees that $\Pi_{k}^{\left(i\right)}=A_{y}^{\left(i\right)}\left(\Sigma_{k}^{y}-Cov\left[{\hat{y}}_{k/k-1}^{\left(i\right)}\right]\right)A_{y}^{(i)T}$. This, together with (22) and the expression of the predictor ${\hat{x}}_{k/k-1}^{\left(i\right)}$, leads to Expression (21), thus completing the proof. □

**Remark** **3.** *Let us observe that the state evolution Equation (1) has not been explicitly used to obtain the proposed filtering algorithm, but only to deduce the expression of the state covariance matrix in a separable form (3), on which the derivation of the algorithm is based. Because of this, the algorithm structure is different from that of most standard algorithms based on the state-space model. Nevertheless, it also admits a prediction-update structure, similar to the conventional Kalman filter, where the filtering estimator and its error covariance matrix are obtained, at each iteration, by updating the prediction estimator and its error covariance, the previous computation of the gain matrix, and the innovation. In fact, from (15)–(21) and the model hypotheses, the following equivalent algorithm is easily derived:*

*For the ith sensor, i=1,…,m, the LS linear filtering estimator ${\hat{x}}_{k/k}^{\left(i\right)}$ and the error covariance matrices $P_{k/k}^{\left(i\right)}$ are obtained from the following recursive algorithm:*

$${\hat{x}}_{k/k}^{\left(i\right)}={\hat{x}}_{k/k-1}^{\left(i\right)}+K_{k}^{\left(i\right)}\mu_{k}^{\left(i\right)},\;\;k\geq 1;\;\;\;\;\;\;\;{\hat{x}}_{0/0}^{\left(i\right)}=0,$$

$$P_{k/k}^{\left(i\right)}=P_{k/k-1}^{\left(i\right)}-K_{k}^{\left(i\right)}\Pi_{k}^{\left(i\right)}K_{k}^{(i)T},\;\;k\geq 1;\;\;\;\;\;\;\;P_{0/0}^{\left(i\right)}=\Sigma_{0}^{x},$$

*where the prediction estimator ${\hat{x}}_{k/k-1}^{\left(i\right)}$ and its error covariance matrices, $P_{k/k-1}^{\left(i\right)},$ satisfy:*

$${\hat{x}}_{k/k-1}^{\left(i\right)}=F_{k-1}{\hat{x}}_{k-1/k-1}^{\left(i\right)},\;\;k\geq 1,$$

$$P_{k/k-1}^{\left(i\right)}=F_{k-1}P_{k-1/k-1}^{\left(i\right)}F_{k-1}^{T}+Q_{k-1},\;\;k\geq 1.$$

*The gain matrix $K_{k}^{\left(i\right)}$ is calculated by:*

$$K_{k}^{\left(i\right)}=\left(F_{k}{\left(D_{k}^{\bar{\theta }}H_{k}B_{k}|C_{k}\right)}^{T}-\left(\Sigma_{k}^{x}-P_{k/k-1}^{\left(i\right)}\right)H_{k}^{T}D_{k}^{\bar{\theta }}\right)\left(I-D_{k}^{\bar{\lambda }}\right)A_{y}^{(i)T}\Pi_{k}^{(i)-1},\;\;k\geq 1.$$

*Finally, the innovations $\mu_{k}^{\left(i\right)}$ and their covariance matrices $\Pi_{k}^{\left(i\right)}$ are obtained as:*

$$\mu_{k}^{\left(i\right)}=A_{y}^{\left(i\right)}\left(y_{k}-\left(I-D_{k}^{\bar{\lambda }}\right)D_{k}^{\bar{\theta }}H_{k}{\hat{x}}_{k/k-1}^{\left(i\right)}\right),\;\;k\geq 1,$$

$$\Pi_{k}^{\left(i\right)}=A_{y}^{\left(i\right)}\left(\Sigma_{k}^{y}-\left(I-D_{k}^{\bar{\lambda }}\right)D_{k}^{\bar{\theta }}H_{k}\left(\Sigma_{k}^{x}-P_{k/k-1}^{\left(i\right)}\right)H_{k}^{T}D_{k}^{\bar{\theta }}\left(I-D_{k}^{\bar{\lambda }}\right)\right)A_{y}^{(i)T},\;\;k\geq 1.$$

### 4.2. Distributed Filtering Estimator Computation

Once the intermediate filtering estimators have been obtained at every sensor *i*, the estimation accuracy can be enhanced by fusing such estimators, ${\hat{x}}_{k/k}^{\left(i\right)}$, with those received from the adjacent nodes, $\left\{{\hat{x}}_{k/k}^{\left(j\right)},\;j\in N_{i}\right\}$. More specifically, as described in Section 3.1, our aim is to design the optimal distributed filter, ${\hat{x}}_{k/k}^{d(i)}$, as the matrix-weighted linear combination of the intermediate estimators available at the *i*th sensor that minimizes the MSE. The following theorem provides the computation routine for such a distributed filter and its error covariance matrix.

**Theorem** **2.** *For the ith sensor, i=1,…,m, the distributed filter, ${\hat{x}}_{k/k}^{d(i)}$, and the error covariance matrices, $P_{k/k}^{d(i)}\equiv Cov\left[x_{k}-{\hat{x}}_{k/k}^{d(i)}\right]$, are obtained by:*

(27) $${\hat{x}}_{k/k}^{d(i)}=G_{k}^{\left(i\right)}A_{x}^{\left(i\right)}{\hat{X}}_{k/k},\;\;\;k\geq 1,$$

(28) $$P_{k/k}^{d(i)}=\Sigma_{k}^{x}-G_{k}^{\left(i\right)}A_{x}^{\left(i\right)}{\left(\Sigma_{k}^{x\hat{X}}\right)}^{T},\;\;\;k\geq 1,$$

*where ${\hat{X}}_{k/k}={\left({\hat{x}}_{k/k}^{(1)T},\ldots ,{\hat{x}}_{k/k}^{(m)T}\right)}^{T}$ and $G_{k}^{\left(i\right)}$ is the minimum MSE weighting matrix, which satisfies:*

(29) $$G_{k}^{\left(i\right)}=\Sigma_{k}^{x\hat{X}}A_{x}^{(i)T}{\left(A_{x}^{\left(i\right)}\Sigma_{k}^{\hat{X}}A_{x}^{(i)T}\right)}^{-1},\;\;\;k\geq 1,$$

*with $\Sigma_{k}^{x\hat{X}}\equiv Cov\left[x_{k},{\hat{X}}_{k/k}\right]$ and $\Sigma_{k}^{\hat{X}}\equiv Cov\left[{\hat{X}}_{k/k}\right]$.*

**Proof.**  According to the considerations made in Section 3.1, the distributed filtering estimator is given by ${\hat{x}}_{k/k}^{d(i)}=G_{k}^{\left(i\right)}{\hat{X}}_{k/k}^{\left(i\right)}$, where ${\hat{X}}_{k/k}^{\left(i\right)}$ is defined in (8); therefore, (27) is proven. Now, taking into account that the estimation error $x_{k}-{\hat{x}}_{k/k}^{d(i)}$ and the estimator ${\hat{x}}_{k/k}^{d(i)}$ are uncorrelated, the error covariance matrix is expressed as $P_{k/k}^{d(i)}=E\left[x_{k}x_{k}^{T}\right]-E\left[{\hat{x}}_{k/k}^{d(i)}x_{k}^{T}\right]$, which, together with (27), provides (28). To complete the proof, we just need to find the optimal weighting matrix, $G_{k}^{\left(i\right)}$, that minimizes the MSE (9), which, as is known, is given by:

$$G_{k}^{\left(i\right)}=Cov\left[x_{k},{\hat{X}}_{k/k}^{\left(i\right)}\right]{\left(Cov\left[{\hat{X}}_{k/k}^{\left(i\right)}\right]\right)}^{-1}.$$

This expression, together with (8), directly yields (29), and the theorem is proven. □

The procedure to obtain the optimal distributed filter established in Theorem 2 requires the calculation of the covariance matrices $\Sigma_{k}^{x\hat{X}}=Cov\left[x_{k},{\hat{X}}_{k/k}\right]$ and $\Sigma_{k}^{\hat{X}}=Cov\left[{\hat{X}}_{k/k}\right].$ Clearly, from its definition, $\Sigma_{k}^{\hat{X}}={\left({\hat{\Sigma }}_{k/k}^{\left(rs\right)}\right)}_{r,s=1,\ldots ,m}$, with ${\hat{\Sigma }}_{k/k}^{\left(rs\right)}\equiv Cov\left[{\hat{x}}_{k/k}^{\left(r\right)},{\hat{x}}_{k/k}^{\left(s\right)}\right]$ and taking into account that the estimation error $x_{k}-{\hat{x}}_{k/k}^{\left(r\right)}$ and the estimator ${\hat{x}}_{k/k}^{\left(r\right)}$ are uncorrelated, we can write $\Sigma_{k}^{x\hat{X}}=\left({\hat{\Sigma }}_{k/k}^{\left(1\right)}\left|\cdots \right|{\hat{\Sigma }}_{k/k}^{\left(m\right)}\right).$ Therefore, the derivation of these covariance matrices is reduced to that of ${\hat{\Sigma }}_{k/k}^{\left(rs\right)}=Cov\left[{\hat{x}}_{k/k}^{\left(r\right)},{\hat{x}}_{k/k}^{\left(s\right)}\right]$, r,s=1,…,m. Next, we propose a recursive algorithm for that purpose. Recursive algorithm for the calculation of ${\hat{\Sigma }}_{k/k}^{\left(rs\right)}$, r,s=1,…,m:

The covariance matrices ${\hat{\Sigma }}_{k/k}^{\left(rs\right)}=Cov\left[{\hat{x}}_{k/k}^{\left(r\right)},{\hat{x}}_{k/k}^{\left(s\right)}\right]$ satisfy:

(30) $${\hat{\Sigma }}_{k/k}^{\left(rs\right)}=F_{k}\Sigma_{k}^{u^{\left(rs\right)}}F_{k}^{T},\;\;k\geq 1,$$

where the matrices $\Sigma_{k}^{u^{\left(rs\right)}}\equiv Cov\left[u_{k}^{\left(r\right)},u_{k}^{\left(s\right)}\right]$ are recursively obtained as follows:

(31) $$\Sigma_{k}^{u^{\left(rs\right)}}=\Sigma_{k-1}^{u^{\left(rs\right)}}+U_{k-1,k}^{\left(rs\right)}\Pi_{k}^{(s)-1}U_{k}^{(s)T}+U_{k}^{\left(r\right)}\Pi_{k}^{(r)-1}\left(U_{k-1,k}^{(sr)T}+\Pi_{k}^{\left(rs\right)}\Pi_{k}^{(s)-1}U_{k}^{(s)T}\right),\;\;k\geq 1;\;\;\;\Sigma_{0}^{u^{\left(rs\right)}}=0,$$

with $U_{k-1,k}^{\left(rs\right)}\equiv Cov\left[u_{k-1}^{\left(r\right)},\mu_{k}^{\left(s\right)}\right]$ and $\Pi_{k}^{\left(rs\right)}\equiv Cov\left[\mu_{k}^{\left(r\right)},\mu_{k}^{\left(s\right)}\right]$ given by:

(32) $$U_{k-1,k}^{\left(rs\right)}=\left(\Sigma_{k-1}^{u^{\left(r\right)}}-\Sigma_{k-1}^{u^{\left(rs\right)}}\right)F_{k}^{T}D_{k}^{\bar{\theta }}H_{k}^{T}\left(I-D_{k}^{\bar{\lambda }}\right)A_{y}^{(s)T},\;\;k\geq 1,$$

(33) $$\Pi_{k}^{\left(rs\right)}=A_{y}^{\left(r\right)}\left(\Sigma_{k}^{y}-\left(I-D_{k}^{\bar{\lambda }}\right)D_{k}^{\bar{\theta }}H_{k}F_{k}\left(\Sigma_{k-1}^{u^{\left(r\right)}}+\Sigma_{k-1}^{u^{\left(s\right)}}-\Sigma_{k-1}^{u^{\left(rs\right)}}\right)F_{k}^{T}H_{k}^{T}D_{k}^{\bar{\theta }}\left(I-D_{k}^{\bar{\lambda }}\right)\right)A_{y}^{(s)T},\;\;k\geq 1.$$

The derivation of (30)–(33) is rather simple, based on the formulas of the intermediate filtering algorithm given in Theorem 1. Actually, from the filter formula (15), Expression (30) is deduced, and (31)–(33) are derived iteratively using the recursive relation (17) and the innovation expression (20).

## 5. Numerical Example

In this section, a numerical simulation example is provided to show the applicability and effectiveness of the proposed distributed filter design scheme for discrete-time stochastic systems with multiplicative noises and fading measurements through sensor networks subject to deception attacks. Namely, the two-dimensional state process ${\left\{x_{k}\right\}}_{k\geq 0}$ is assumed to be described by the following model:

$$x_{k+1}=\left(F+\alpha_{k}\overset{˘}{F}\right)x_{k}+Gw_{k},\;\;\;\;k\geq 0,$$

where

$$F=\left(\begin{array}{cc} \;0.95 & 0.01\\ \;0 & 0.95 \end{array}\;\right),\;\;\;\overset{˘}{F}=\left(\begin{array}{cc} \;0.01 & 0\\ \;0 & 0.01 \end{array}\;\right),\;\;\;G=\left(\begin{array}{c} \;0.8\\ \;0.6 \end{array}\;\right).$$

The initial state, $x_{0}$, is a two-dimensional standard normal random vector. and ${\left\{\alpha_{k}\right\}}_{k\geq 0}$, ${\left\{w_{k}\right\}}_{k\geq 0}$ are zero-mean Gaussian white scalar processes with unit variance. These noise sequences and the initial state are assumed to be mutually independent; then, it is clear that the state covariance function can be expressed in a separable form as $Cov\left[x_{k},x_{l}\right]=F_{k}B_{l}^{T}$, with $F_{k}=F^{k}$ and $B_{l}^{T}=F^{-l}\Sigma_{l}^{x}$, where $\Sigma_{l}^{x}=Cov\left[x_{l}\right]$ is recursively obtained by:

$$\Sigma_{l}^{x}=F\Sigma_{l-1}^{x}F^{T}+\overset{˘}{F}\Sigma_{l-1}^{x}{\overset{˘}{F}}^{T}+GG^{T}\;,\;\;\;h\geq 1;\;\;\;\;\Sigma_{0}^{x}=I.$$

For the simulation, let us consider that the sensor network has the same topological structure as that in [25], represented by a digraph G=(V,E,A), with set of nodes V={1,2,3,4}, set of edges $E=\left\{\;(1,1),(1,2),(1,3),(2,2),(2,3),(2,4),(3,1),(3,3),(3,4),(4,1),(4,2),(4,4)\;\right\}$, and binary adjacency matrix $A={\left(a_{ij}\right)}_{m\times m}$, such that $a_{ij}=1$ if and only if (i,j)∈E and $a_{ij}=0$ otherwise.

According to the theoretical study, we consider that the observations in the four sensor nodes are affected by different uncertainties; namely, we suppose that the measured outputs of the four sensor nodes are described by (2), where the model parameters and noises are chosen as follows:

- $H_{k}^{\left(1\right)}=\left(0.8,\;0.9\right),\;\;H_{k}^{\left(2\right)}=\left(0.6,\;0.7\right),\;\;H_{k}^{\left(3\right)}=\left(0.7,\;0.8\right),\;\;H_{k}^{\left(4\right)}=\left(0.9,\;0.5\right)$, and ${\overset{˘}{H}}_{k}^{\left(1\right)}={\overset{˘}{H}}_{k}^{\left(2\right)}={\overset{˘}{H}}_{k}^{\left(3\right)}=\left(0,\;0\right)$, ${\overset{˘}{H}}_{k}^{\left(4\right)}=\left(0,\;0.95\right).$
- ${\left\{\theta_{k}^{\left(i\right)}\right\}}_{k\geq 1},\;i=1,2,3,4$, and ${\left\{\beta_{k}^{\left(4\right)}\right\}}_{k\geq 1}$ are mutually independent sequences of independent random variables with the following time-invariant probability distributions:
  – $\theta_{k}^{\left(1\right)}$ is uniformly distributed over [0.3,0.7] (continuous fading measurements in Sensor 1).
  – $P\left[\theta_{k}^{\left(2\right)}=0\right]=0.1,\;\;P\left[\theta_{k}^{\left(2\right)}=0.5\right]=0.5,\;\;P\left[\theta_{k}^{\left(2\right)}=1\right]=0.4$ (discrete fading measurements in Sensor 2).
  – $\theta_{k}^{\left(3\right)}$ and $\theta_{k}^{\left(4\right)}$ are Bernoulli random variables with $P\left(\theta_{k}^{\left(i\right)}=1\right)=\bar{\theta }$, for i=3,4 (missing measurements in Sensors 3 and 4).
  – $\beta_{k}^{\left(4\right)}$ is a standard Gaussian variable (measurement multiplicative noise in Sensor 4).
  Note that the missing measurements in the third and fourth sensors are obviously extreme cases of the fading ones, so they can be covered by the current approach.
- The additive sensor noises ${\left\{v_{k}^{\left(i\right)}\right\}}_{k\geq 1}$, i=1,2,3,4, are defined by $v_{k}^{\left(i\right)}=v_{i}w_{k-1}$, with $v_{1}=v_{4}=25$ and $v_{2}=v_{3}=50$. Clearly, these noises are correlated to each other, with $R_{k}^{\left(ij\right)}=v_{i}v_{j}$, and also correlated with the process noise, with $S_{k,k+1}^{\left(i\right)}=Gv_{i}$. Hence, the state process and the sensor noises are correlated, and $C_{k}^{(i)T}=F^{-k}Gv_{i}.$

Furthermore, in line with the theoretical study, assume that the measurements of the sensor nodes are subject to deception attacks and the signal injected by the adversaries is given by (5). The false data injection attack noise, $\varepsilon_{k}^{\left(i\right)}$, is chosen as $\varepsilon_{k}^{\left(i\right)}=e_{i}\varepsilon_{k}$, with $e_{i}=0.25i$, i=1,2,3,4, and ${\left\{\varepsilon_{k}\right\}}_{k\geq 1}$ a standard Gaussian white process; hence, these noises are correlated with $T_{k}^{\left(ij\right)}=e_{i}e_{j}$.

The measurements for the estimation are described by (6), where the white sequences of Bernoulli random variables ${\left\{\lambda_{k}^{\left(i\right)}\right\}}_{k\geq 1}$, i=1,2,3,4, modeling whether the deception attacks actually happen or not, are identically distributed with probabilities $P\left(\lambda_{k}^{\left(i\right)}=1\right)={\bar{\lambda }}^{\left(i\right)}$.

In order to show the effectiveness of the filtering algorithms presented in Theorems 1 and 2 and to quantify the estimation accuracy, the error variances of the proposed filters were calculated at every sensor node *i*, for i=1,2,3,4. Different probabilities $\bar{\theta }$, that the signal is present in the measured outputs of Sensors 3 and 4, were considered to show the effect of the missing measurement phenomenon on the performance of the proposed distributed estimators, and different success probabilities ${\bar{\lambda }}^{\left(i\right)}$ of attacks were also chosen to analyze how these probabilities influence the distributed filtering error variances.

**Figure 1:**
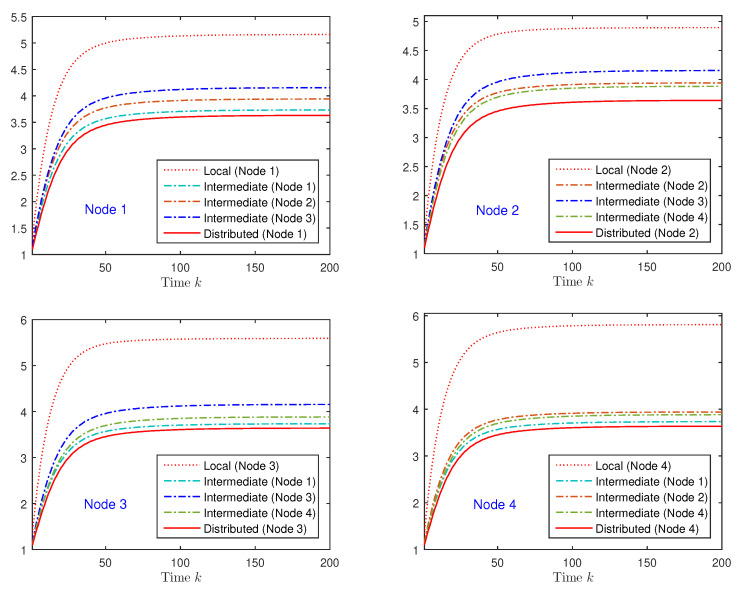
Error variance comparison of the local, intermediate, and distributed filtering estimators for the first component of the state vector.

Considering that $\bar{\theta }=0.75$ and choosing the attack probabilities as ${\bar{\lambda }}^{\left(1\right)}={\bar{\lambda }}^{\left(2\right)}=0.6$ and ${\bar{\lambda }}^{\left(3\right)}={\bar{\lambda }}^{\left(4\right)}=0.7$, Figure 1 shows, for the first state component, the error variances of the local filter (obtained using only the measurements from the *i*th sensor itself) and those of the proposed intermediate and distributed filters at every sensor node *i*. From Figure 1, it is observed, on the one hand, that the error variance corresponding to the intermediate filter is significantly less than that of the local filter and, on the other, that the distributed filter outperforms all the intermediate filters in its neighborhood. Therefore, at each sensor node *i*, the performance of the local filter is considerably improved by using not only the measurements of the sensor itself, but also those coming from its neighbor nodes (proposed intermediate filter), and this performance is further improved by fusing the intermediate filters obtained in the sensor neighborhood (proposed distributed filter). Similar results are obtained for the second component of the state vector.

**Figure 2:**
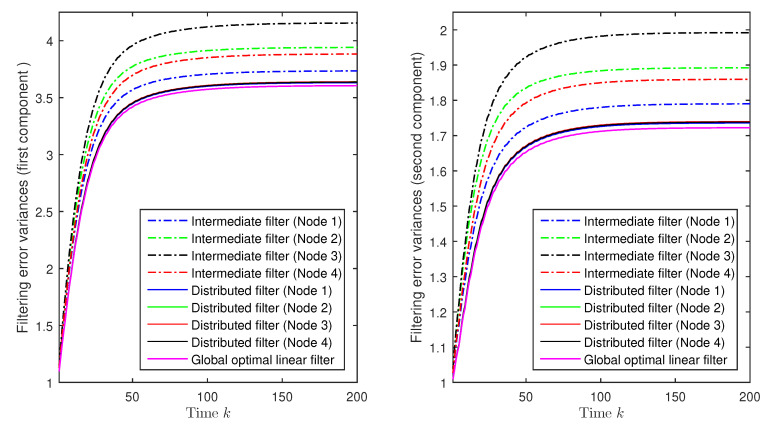
Filtering error variance comparison of intermediate, distributed, and global optimal linear filters.

Furthermore, it is evident from Figure 1 that, even when the same type of estimators (local, intermediate, or distributed) is considered, the results are different from one node to another, which was expected since they are based on different sets of measured data. A desirable property in the design of estimators over sensor networks is that such discrepancies between sensors are as small as possible; for a more explicit visualization of these differences, Figure 2 displays, for the first and second state components, the error variances of the intermediate and the distributed filters in the four nodes, as well as those of the global optimal linear filter, based on the complete set of measurements coming from all the network nodes. As we can see from this figure, the distance between the error variances of the distributed filters in the different nodes is significantly smaller than that between the intermediate ones, which, additionally, is close to the global optimal error variances, meaning that not only do the proposed distributed filters present less discrepancies than the intermediate ones, but also, they show a tightly similar performance to that of the global optimal filter. Therefore, we can conclude that the accuracy of the proposed distributed filtering estimators is satisfactory under missing measurements and deception attacks.

**Figure 3:**
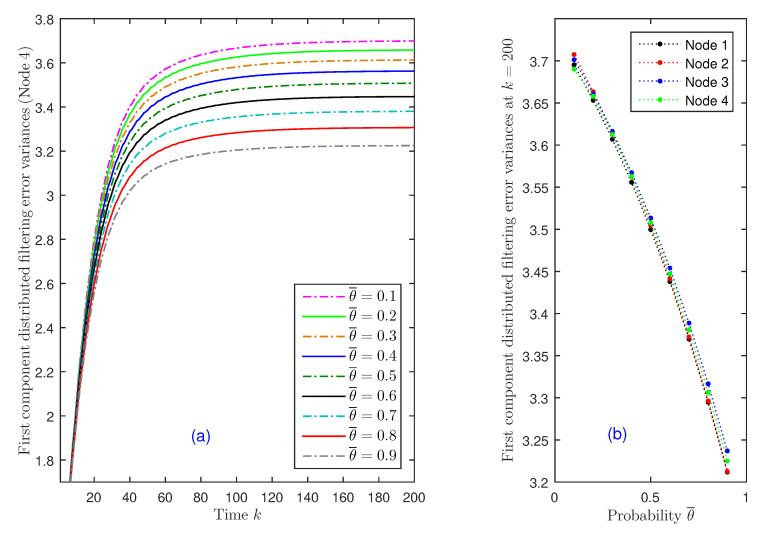
Distributed filtering error variances: (**a**) for $\bar{\theta }=0.1$ to 0.9, in Node 4; (**b**) at k=200, versus $\bar{\theta }$, in all nodes.

**Figure 4:**
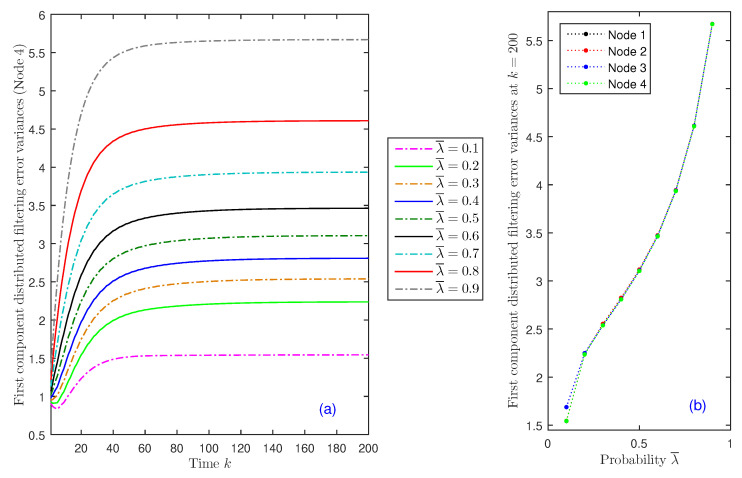
Distributed filtering error variances: (**a**) for $\bar{\lambda }=0.1$ to 0.9, in Node 4; (**b**) at k=200, versus $\bar{\lambda }$, in all nodes.

Next, the effect of the missing measurement and attack probabilities on the performance of the distributed filtering estimators is examined in terms of the error variances. Since similar results are obtained for the two components of the state vector, only those corresponding to the first one are presented.

(I) Impact of the missing measurement phenomenon: Assuming, as in Figure 1, that the probabilities of attacks are ${\bar{\lambda }}^{\left(1\right)}={\bar{\lambda }}^{\left(2\right)}=0.6$ and ${\bar{\lambda }}^{\left(3\right)}={\bar{\lambda }}^{\left(4\right)}=0.7$, the effect of the missing measurement phenomenon is studied by analyzing how the distributed filtering error variances are influenced by the probability $\bar{\theta }$ that the signal is present in the measured outputs of Sensors 3 and 4. First, Figure 3a shows the distributed error variances for the values $\bar{\theta }=0.1$ to 0.9, at Sensor Node 4. From this figure, it is observed that the performance of the distributed filtering estimators is indeed influenced by the value of $\bar{\theta }$, and as expected, the error variances are smaller as the values of the probability $\bar{\theta }$ increase (or, equivalently, when the missing measurement probability, $1-\bar{\theta }$, decreases). Similar results are obtained for the rest of sensor nodes in the network. Indeed, taking into account that the behavior of the distributed filtering error variances is analogous in all the iterations, for a better visualization of this decreasing trend in all the sensor nodes, Figure 3b displays the distributed error variances in the four nodes, only at iteration k=200.

(II) Impact of deception attacks: Considering now that $\bar{\theta }=0.75$ is fixed, we analyze the impact of the deception attacks on the estimation accuracy. For this purpose, we assume that the probability of successful attack is the same for the four sensors, ${\bar{\lambda }}^{\left(i\right)}=\bar{\lambda }$, i=1,2,3,4, and we compare the distributed filtering error variances for different values of this probability, $\bar{\lambda }=0.1$ to 0.9. The results for Sensor Node 4 are displayed in Figure 4a, which shows, as expected, that the performance of the distributed filter becomes worse as the attack probabilities, $\bar{\lambda }$, increase. Similar results are obtained in all nodes, as one can observe in Figure 4b, which displays the distributed filtering error variances at k=200 versus $\bar{\lambda }$ in the four sensor nodes. This figure shows that, in fact, the behavior is analogous in the different nodes and also that the growth of the error variances is more evident for higher values of $\bar{\lambda }$.

**Figure 5:**
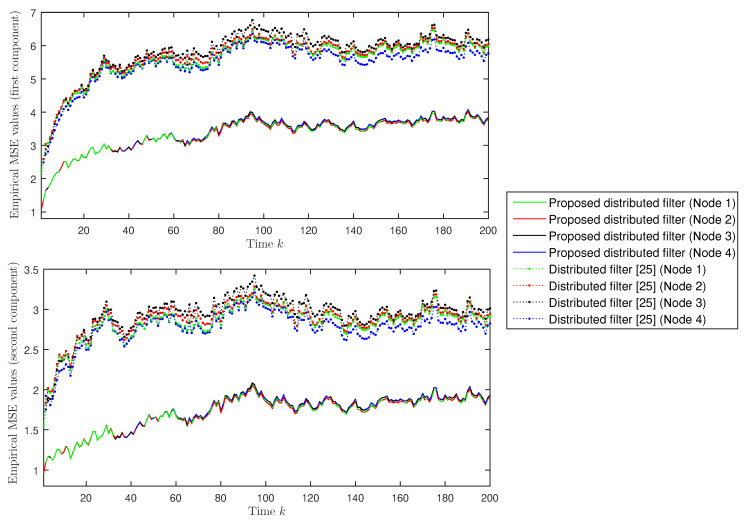
Empirical mean squared error comparison of the proposed distributed filter and the distributed filter in [25].

Finally, we present a comparative analysis of the proposed distributed filter and the distributed filter [25] for networked systems with random parameter matrices and correlated noises. Considering the same probabilities ($\bar{\theta }=0.75$, ${\bar{\lambda }}^{\left(1\right)}={\bar{\lambda }}^{\left(2\right)}=0.6$ and ${\bar{\lambda }}^{\left(3\right)}={\bar{\lambda }}^{\left(4\right)}=0.7$) as in Figure 1 and using one thousand independent simulations, both filtering estimates are compared on the basis of the empirical mean squared error values, which are calculated as:

$${\mathrm{MSE}}_{k}^{\left(i,j\right)}=\frac{1}{1000}\sum_{s=1}^{1000}{\left(x_{k}^{\left(j,s\right)}-{\hat{x}}_{k/k}^{d(i,j,s)}\right)}^{2},\;\;\;1\leq k\leq 200,\;\;i=1,2,3,4,\;\;j=1,2,$$

where $x_{k}^{\left(j,s\right)}$ denotes the *j*th component of the simulated state and ${\hat{x}}_{k/k}^{d(i,j,s)}$ is the *j*th component of the filter calculated in the *i*th sensor node, at the sampling time *k*, in the *s*th simulation run. The results are displayed in Figure 5, which shows that, for both the first and second components, the proposed distributed filter outperforms the filter in [25]. This fact could be expected, since the proposed distributed filter accommodates the simultaneous effect of multiplicative noise, fading measurements, and stochastic deception attacks in the different sensors, while the filter in [25] does not take into account the stochastic deception attack phenomenon.

## 6. Conclusions

In this paper, a general theoretical framework is established to address the optimal distributed filtering problem in discrete-time stochastic multi-sensor systems suffering random uncertainties, including fading measurements and multiplicative noises, under deception attacks. The fading phenomenon is modeled by [0,1]-valued random variables, thus covering the possibility of missing measurements as a particular case. The spatial distribution of the sensors is represented by a known digraph, and at every sensor node, the proposed distributed filtering technique operates in two phases. In the first phase, an intermediate optimal linear estimator is obtained, using its own local measurements and those received from its adjacent nodes, while the second phase consists of fusing the sensor intermediate filter with those calculated by its adjacent nodes to obtain the desired distributed filter as the minimum mean squared error matrix-weighted linear combination of the intermediate estimators. It is noticeable that the derivation of the proposed filtering technique does not require the explicit information provided by the state evolution equation itself, but only the factorization of the state covariance matrix in a separable form (3). Because of this, the proposed distributed filtering technique can be applied to estimate stationary or non-stationary signals whose covariance matrix can be factorized in this form, regardless of the fact that the signal evolution model is fully known or not. Finally, a simulation example shows the satisfactory performance of the proposed filtering scheme and illustrates the relation between the estimation accuracy and the success probability of attacks.
